# Genetic Analysis of *Prunus salicina* L. by Random Amplified Polymorphic DNA (RAPD) and Intersimple Sequence Repeat (ISSR)

**DOI:** 10.1155/2022/2409324

**Published:** 2022-04-18

**Authors:** Jun Li, Guangchun Gao, Bin Li, Bai Li, Qihua Lu

**Affiliations:** ^1^College of Modern Agriculture, Jiaxing Vocational and Technical College, Jiaxing, Zhejiang 314000, China; ^2^College of Medicine, Jiaxing University, Jiaxing, Zhejiang 314001, China; ^3^Jiaxing City General Station of Cropping Technical Extension, Jiaxing, Zhejiang 314000, China; ^4^Jiaxing Academy of Agricultural Sciences, Jiaxing, Zhejiang 314016, China; ^5^Horticultural Crop and Plum Research Institute, Jiaxing, Zhejiang 314016, China

## Abstract

**Background:**

*Prunus salicina* L. is an important fruit tree species of great economic value which is mainly distributed in the northern hemisphere.

**Methods:**

25 samples of *Prunus salicina* L. were collected from 8 provinces in China, Japan, USA, and New Zealand. The genetic variations of these samples were characterized by the random amplified polymorphic DNA (RAPD) and intersimple sequence repeat (ISSR) technique, respectively, and in combination.

**Results:**

Totally, 257 RAPD bands ranging 200∼2300 bp was found, and 81.59% of these bands were polymorphic. ISSR analysis identified 179 bands ranging 300∼2500 bp, and 87.74% of the bands were polymorphic. ISSR results showed that the similarity coefficient index between samples P10 (Maihuangli in Anhui, Chin) and P13 (Longyuanqiuli in Heilongjiang, China) was lowest, while that between samples P10 (Maihuangli in Anhui, Chin) and P15 (Baili in Japan) was highest. Combined analysis of RAPD and ISSR demonstrated that the similarity coefficient index between samples P4 (Qiepili in Ningbo, Zhejiang, China) and P13 (Longyuanqiuli in Heilongjiang, China) was lowest, while that between samples P19 (Laroda in USA) and P20 (Red heart in USA) was highest.

**Conclusion:**

RAPD combined with ISSR analysis can be used for genetic characterization of *Prunus* L. species.

## 1. Background

*Prunus salicina* L., belonging to the family of Rosaceae, are one of the most important economical fruit trees and are widely cultivated all over the world. They are mainly distributed in the northern hemisphere, especially in the temperate zone [1, 2]. China is one of the origin and distribution centers of *Prunus* L. species. *Prunus* L. species contain more than 430 species and are first segregated into six genera according to the morphology of fruit: *Amygdalus* L., *Armeniaca* Scop., *Cerasus* Mill., *Laurocerasus*, *Padus* Mill., *Prunus* species, and Tourn. ex Duh. However, phylogenetic analysis showed that *Cerasus*, *Laurocerasus*, and *Padus* were not monophyletic [3, 4]. Besides, an increasing number of new cultivars from different countries result an important renewal of plant material worldwide [2]. It is thus necessary to characterize genetic information of *Prunus* L. species to cultivate new breed with improved quality characteristics.

DNA polymorphism assay based on the amplification of random DNA segments with single primers of arbitrary nucleotide sequence has been widely used for genetic diversity analysis of species [5]. Several studies have been devoted to the genetic diversity in *Prunus* L. species [6–8]. Recently, a number of molecular marker techniques including random amplified polymorphic DNA (RAPD), simple sequence repeat (SSR), intersimple sequence repeat (ISSR), and amplified fragment length polymorphism (AFLP) have been developed and widely used in the identification of various organisms [4, 6, 9–11]. Among these techniques, RAPD and ISSR methods are two PCR-based methods that require only small amounts of DNA sample without involving radioactive labels and therefore have been widely used for genetic characterization [12]. RAPD is a technique based on the amplification of the genomic DNA with either a single or multiple short oligonucleotide primers of an arbitrary or random sequence [12]. RAPD is simple, cost-efficient, and does not require DNA sequences before application [13]. ISSR is derived from SSR, which is more abundant, informative, highly polymorphic, and efficient [14]. RAPD and ISSR methods have separately been used for genetic characterization in many species, such as *Lonicera japonica* Thunb. [15], synthetic hexaploid wheats [16], *Atractylodes lancea* [17], and *Ocimum basilicum* L. [18]. However, because of their advantages and disadvantages, more studies applied RAPD combined with ISSR to characterize the genetic variation of species, such as *Litchi chinensis* Sonn. [19], *Allium* species [20], date palm [21], and *Cymbopogon* [22]. However, only limited studies have been conducted to characterize the genetic relationships among different genus or cultivars of *Prunus* L. species [23–25].

In this study, we applied the RAPD and ISSR technique for the genetic characterization of 25 *P*. *salicina* from China and other countries. This study may provide valuable insight into the genetic diversity of *P*. *salicina* L. and provide information to cultivate new breed with improved traits.

## 2. Methods

### 2.1. Plant Material Collection and DNA Extraction

**Figure 1:**
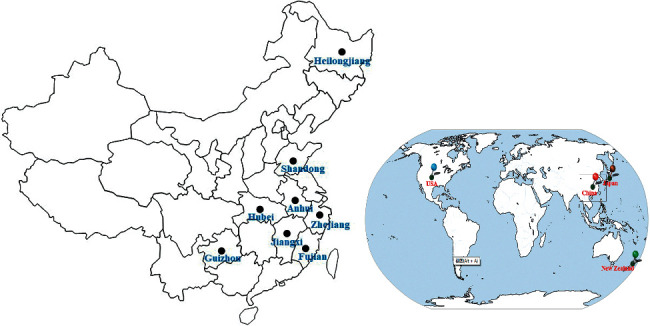
The localities of samples of *P*. *salicina* L. from different regions. The spots in black indicate the provinces in China.

**Figure 2:**
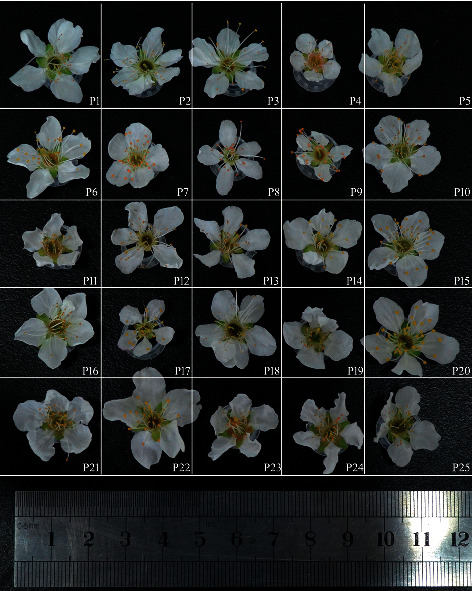
The flowers of 25 *P*. *salicina* L.

This study included 25 *P*. *salicina* L. which were collected from 14 different regions from China (13 samples), Japan (4 samples), USA (7 samples), and New Zealand (1 sample) (Figure 1 and Table 1). Among them, P1, P2, and P3 are the three lines with different maturity of one cultivar. The flowers of the 25 *P*. *salicina* L. are shown in Figure 2.

The genomic DNA of 25 *P*. *salicina* L. was extracted from fresh leaves using a modified cetyl trimethylammonium bromide (CTAB) method as described previously [15, 26]. DNA integrity was checked by 0.8% agarose gel electrophoresis, and DNA purity was determined by the absorbance ratio at 260 nm : 280 nm on spectrophotometry. The final concentration of DNA samples was adjusted to 10 ng/*µ*l for PCR and stored at −20°C until use.

### 2.2. Amplification of DNA by RAPD-PCR

The random RAPD primers were selected randomly for PCR amplification (Table 2). The PCR system in 10 *μ*L volume contains 1 *μ*L of 2.5 *μ*mol/L primers, 1 *μ*L of DNA template, 5 *μ*L of 2 × PCR Taq Mastermix (TianGen Biotech Co. Ltd., Beijing), and 3 *μ*L of deionized water. The PCR was executed on Applied Biosystems Veriti 96-Well Thermal Cycler (Thermo Fisher, USA) in the following procedure: initial denaturation at 95°C for 90 s, followed by 40 cycles of 40 s at 94°C, 60 s at 36°C, 90 s at 72°C, and final extension of 5 min at 72°C.

### 2.3. ISSR Amplification

Fifteen ISSR primers were synthesized by Thermo Fisher (USA) (Table 2). ISSR amplification was performed in 10 *μ*L reactions including 1 *μ*L of 2.5 umol/L primers, 1 *μ*L of DNA template, 5 *μ*L of 29 PCR Taq Mastermix (TianGen Biotech Co. Ltd., Beijing), and 3 *μ*L of deionized water. PCR was executed on Applied Biosystems Veriti 96-Well Thermal Cycler using the following procedure: initial denaturation at 95°C for 90 s, followed by 35 cycles of 30 s at 94°C, 30 s at 50°C, 90 s at 72°C, and final extension of 5 min at 72°C [15].

### 2.4. Agarose Gel Electrophoresis

The amplified PCR products were separated by electrophoresis on 1.8% agarose gel in 1 × TAE buffer. Gels were visualized by 0.5 g/ml ethidium bromide staining, and the images were documented using the ChemiDoc XR (Bio-Rad, USA). Bands that were unambiguous and reproducible in successive amplifications were selected for scoring.

### 2.5. Data Analysis

All PCRs were repeated five times for each of five samples. Bands in the gel profiles were scored as 1 for present and 0 for absent. The similarity matrix (SM) and the similarity index (SI) were calculated using SM coefficient in Numerical Taxonomy Multivariate Analysis System (NTSYS pc 2.1) software. The dendrogram based on the unweighted pair group method with arithmetic mean algorithm (UPGMA) was generated using the SAHN module in the NTSYS pc 2.1 software.

## 3. Results

### 3.1. Amplification of DNA by RAPD and ISSR

**Figure 3:**
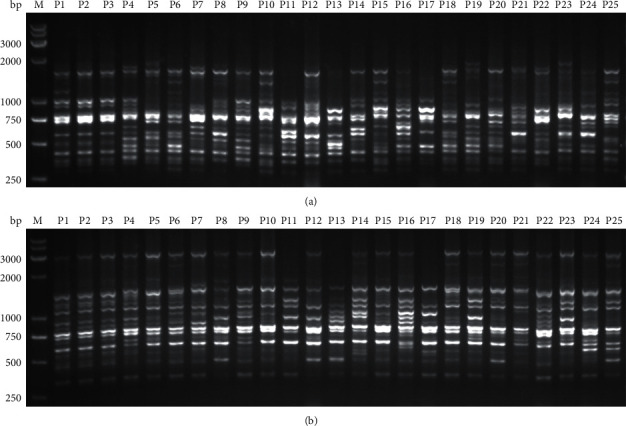
The representative results of banding profiles obtained by ISSR primer UBC807 (a) and RAPD primer S201 (b). Lanes P1–P25 represented different samples listed in Table 1. Lane “M” represents the DL2000 DNA marker.

A total of nineteen RAPD primers and fifteen ISSR primers were used in this study for the evaluation of DNA polymorphism (Table 2). All RAPD primers and ISSR primers generated evaluable bands.  Figure 3 shows the representative reproducible polymorphic amplification bands in these 25 samples generated from ISSR primer UBC807 and RAPD primer S201. For the RAPD primers, a total of 315 bands with an average of 16.58 bands per primer were obtained. Among these bands, 257 (81.59%) bands were polymorphic, and the approximate band size ranged from 200 bp to 2300 bp. The minimum number of bands was 10, which was generated by primer OPA-4 and the maximum was 21, which was produced by primer S43. The total number of polymorphic fragments ranged from 7 (primer OPA-4) to 18 (primer OPA-10). The average polymorphic fragments ratio (PFR) (in %) was 81.60% (min: 65%; max: 94.74%). The other information of the bands generated by RAPD primers, including polymorphism information content (PIC), resolving power (RP), effective multiplex ratio (EMR), and marker index (MI), are presented in Table 3.

For the ISSR primers, a total of 204 bands with an average of 13.60 bands per primer were produced; of them, 179 (87.74%) were polymorphic. The approximate range of band size was 300 bp to 2500 bp (Table 4). The minimum number of bands was 8, which was yielded by primer UBC829, and the maximum was 19, which was produced by primer UBC807. The total number of PFs ranged from 8 (primer UBC829) to 15 (primers UBC807, UBC810, UBC846, and UBC881). The average PFR% was 87.80% (min: 69.23%; max: 100%). The other information of the bands generated by ISSR primers, including PIC, RP, EMR, and MI, are presented in Table 4.

### 3.2. Genetic Distance and Cluster Analysis of RAPD and ISSR Markers

**Figure 4:**
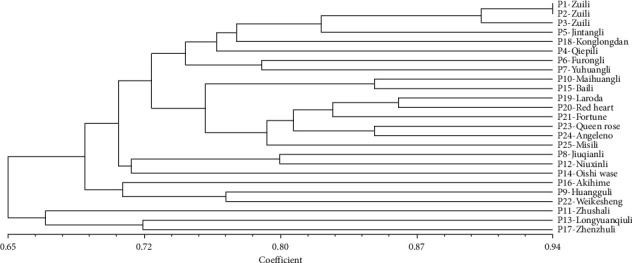
Dendrogram of cluster of 25 *P*. *salicina* L. based on RAPD markers.

Based on the RAPD amplification profiles, cluster dendrogram was obtained using UPGMA (Figure 4). Since P1, P2, and P3 belong to one cultivar, we ignored their coefficients in the following analysis. The dendrogram showed that the similarity coefficients ranged from 0.584 to 0.860. In the RAPD-based dendrogram, the 25 *P*. *salicina* samples formed four clusters at a cutoff of 0.692. The similarity coefficient between sample P4 (Qiepili in Ningbo, Zhejiang, China) and P13 (Longyuanqiuli in Heilongjiang, China) was lowest (0.584), while that between sample P19 (Laroda in USA) and P20 (Red heart in USA) was highest (0.860) (Figure 4).

**Figure 5:**
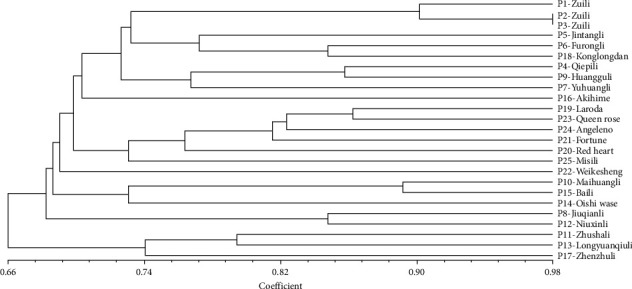
Dendrogram of cluster of 25 *P*. *salicina* L. based on ISSR markers.

The ISSR analysis showed similar results to the RAPD analysis. The dendrogram showed that the similarity coefficients ranged from 0.558 to 0.892. In the ISSR-based dendrogram, the 25 *P. salicina* samples were divided into five clusters at a cutoff of 0.692. The similarity coefficient between sample P10 (Maihuangli in Anhui, China) and P13 (Longyuanqiuli in Heilongjiang, China) was lowest (0.558), while that between sample P10 (Maihuangli in Anhui, Chin) and P15 (Baili in Japan) was highest (0.892) (Figure 5).

### 3.3. Integrating Analysis of RAPD and ISSR Data

The dendrogram results of RAPD combined with ISSR showed that the similarity coefficients ranged from 0.597 to 0.865. Total 519 DNA fragments were yielded, of which 435 (84.7%) were polymorphic. The average number of PF per primer was 12.7. The mean PIC, RP, EMR, and MI values observed for all primers were 0.42, 17.77, 10.80, and 4.54, respectively (Table 5). The similarity coefficients between sample P10 (Maihuangli in Anhui, China) and P13 (Longyuanqiuli in Heilongjiang, China) was lowest (0.597), while that between sample P10 (Maihuangli in Anhui, China) and P15 (Baili in Japan) was highest (0.865) (Table 6).

### 3.4. Typical Band Patterns Amplified by ISSR and RAPD Markers

**Figure 6:**
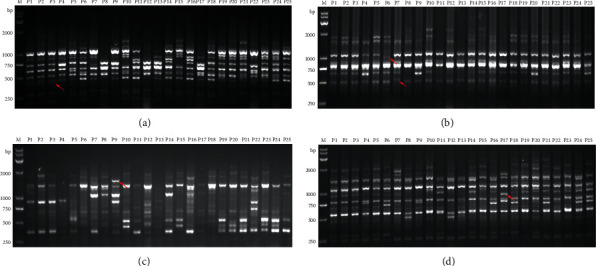
The representative results of banding profiles obtained by ISSR primers UBC834 (a), UBC847 (b), UBC857 (c), and RAPD primer S1403 (d). Lanes P1–P25 represent different samples listed in Table 1. Lane “M” represents the DL2000 DNA marker. The typical bands for molecular identification of *P*. *salicina* L. are indicated by a red arrow.

Sixteen primers, including 11 ISSR primers and 5 RAPD primers, could be used as the markers of molecular identification for 25 *Prunus* L. samples (Table 7). As shown in Table 7, UBC810, UBC834, and UBC836 could be considered as the markers of P1 (Zuili1 in Jiaxing, Zhejiang, China), P2 (Zuili2 in Jiaxing, Zhejiang, China), and P3 (Zuili3 in Jiaxing, Zhejiang, China). S17 could be considered as a marker of P4 (Qiepili in Ningbo, Zhejiang, China). UBC881 might be a marker of P5 (Jintangli in Zhoushan, Zhejiang, China). UBC847 was a marker of P6 (Furongli in Fujian, China). UBC847 and UBC855 could be used to distinguish P7 (Yuhuangli in Hubei, China). UBC848 could be considered as a marker of P8 (Jiuqianli in Guizhou, China). UBC857 might be a potential marker of P9 (Huangguli in Tongxiang, Zhejiang, China). RAPD-1 could be used as a marker of P12 (Niuxinli in Shandong, China). UBC889 could be considered as a marker of P16 (Akihime in Japan). S43 and S1403 might be the markers of P17 (Zhenzhuli in Japan). UBC829 might be a potential marker of P23 (Queen rose in USA). RAPD-5 also might be used a marker of P25 (Misili in New Zealand). The representative banding profiles obtained by ISSR primers UBC834, UBC847, UBC857, and RAPD primer S1403 are shown in Figure 6.

## 4. Discussion

Illustration of the genetic relationships or characterization of genetic diversity is important to provide genetic guidance for hybrid breeding. In this study, the genetic diversity and relationship among 25 *P. salicina* L. varieties were evaluated by RAPD and ISSR, respectively, and integrated. The Dice's similarity coefficient of RAPD ranged from 0.584 to 0.860, and that of ISSR ranged from 0.558 to 0.892. Integrating analysis of RAPD and ISSR indicated the similarity coefficient varied from 0.597 to 0.865. The results indicated high diversity among the 25 varieties.

ISSR and RAPD were widely used for genetic diversity evaluations of *Prunus* L. species. Tian et al. used ISSR and RAPD for genetic diversity evaluations of 48 *Prunus mira* L. samples, the high levels of polymorphism, and the results imply that Tibet samples preserved higher genetic diversity and most genetic variations occurred [27]. However, the efficiency of RAPD markers and ISSR markers in detecting polymorphism is controversial. Tian et al. demonstrated that ISSR found 77.80% polymorphism, which is higher than that found by RAPD (72.73%). In the study of Kumar et al. the phylogenetic relationships of 36 locally grown *P. armeniaca* genotypes were analyzed using 20 RAPDs and 11 ISSRs markers. RAPD markers were found more efficient for polymorphism detection, as they detected 97.84% as compared to 96.5% for ISSR markers, and the pattern of clustering of the genotypes remained more or less the same in RAPD and combined data of RAPD + ISSR [28]. In our study, the PFR% of RAPD primers was 81.60%, which is lower than that of ISSR primers (87.80%). Our results support the view that ISSR markers are more efficient than RAPD with regards to detecting polymorphism.

The RAPD results showed that the index of similarity coefficient between sample P4 (Qiepili in Ningbo, Zhejiang, China) and P13 (Longyuanqiuli in Heilongjiang, China) was lowest (0.584), while that between sample P19 (Laroda in USA) and P20 (Red heart in USA) was highest (0.860). However, the ISSR results showed that the index of similarity coefficient between sample P10 (Maihuangli in Anhui, Chin) and P13 (Longyuanqiuli in Heilongjiang, China) was lowest (0.558), while that between sample P10 (Maihuangli in Anhui, China) and P15 (Baili in Japan) was highest (0.892). In addition, the analysis of RAPD combined with ISSR showed that the similarity coefficient between sample P10 (Maihuangli in Anhui, China) and P13 (Longyuanqiuli in Heilongjiang, China) was lowest (0.597), while that between sample P10 (Maihuangli in Anhui, Chin) and P15 (Baili in Japan) was highest (0.865), which was consistent with the RAPD analysis. These findings demonstrated that the RAPD technique not only increased the resolution and yield but also was a reliable molecular tool for the genetic characterization of various organisms, which was reported in previous studies [6, 15]. Our RAPD and ISSR analysis showed potentiality to distinguish *P. salicina* L. from related genus or species.

## 5. Conclusion

In summary, our study indicates that the RAPD combined with ISSR techniques would be used for the genetic diversity, molecular-assisted breeding, and genetic characterization of *P. salicina* L. Our results might assist in parental gametophytes selection for hybrid breeding of *P. salicina* L.

## Figures and Tables

**Table 1:** Sources of RAPD and ISSR samples.

Sample number	Cultivars	Species	Origin
P1	Zuili1	*P. salicina*	Jiaxing, Zhejiang, China
P2	Zuili2	*P. salicina*	Jiaxing, Zhejiang, China
P3	Zuili3	*P. salicina*	Jiaxing, Zhejiang, China
P4	Qiepili	*P. salicina*	Ningbo, Zhejiang, China
P5	Jintangli	*P. salicina*	Zhoushan, Zhejiang, China
P6	Furongli	*P. salicina*	Fujian, China
P7	Yuhuangli	*P. salicina*	Hubei, China
P8	Jiuqianli	*P. salicina*	Guizhou, China
P9	Huangguli	*P. salicina*	Tongxiang, Zhejiang, China
P10	Maihuangli	*P. salicina*	Anhui, China
P11	Zhushali	*P. salicina*	Jiangxi, China
P12	Niuxinli	*P. salicina*	Shandong, China
P13	Longyuanqiuli	*P. salicina* hybrid	Heilongjiang, China
P14	Oishi wase	*P. salicina*	Japan
P15	Baili	*P. salicina*	Japan
P16	Akihime	*P. salicina*	Japan
P17	Zhenzhuli	*P. salicina*	Japan
P18	Konglongdan	*P. salicina* hybrid	USA
P19	Laroda	*P. salicina* hybrid	USA
P20	Red heart	*P. salicina* hybrid	USA
P21	Fortune	*P. salicina* hybrid	USA
P22	Weikesheng	*P. salicina* hybrid	USA
P23	Queen rose	*P. salicina* hybrid	USA
P24	Angeleno	*P. salicina* hybrid	USA
P25	Misili	*P. salicina*	New Zealand

**Table 2:** Sequences of ISSR and RAPD primers.

Primer	Sequence (5′-3′)	Primer	Sequence (5′-3′)
ISSR			
UBC807	AGAGAGAGAGAGAGAGT	UBC810	GAGAGAGAGAGAGAGAT
UBC826	ACACACACACACACACC	UBC827	ACACACACACACACACG
UBC829	TGTGTGTGTGTGTGTGC	UBC834	AGAGAGAGAGAGAGAGYT
UBC836	AGAGAGAGAGAGAGAGYA	UBC846	CACACACACACACACART
UBC847	CACACACACACACACARC	UBC848	CACACACACACACACARG
UBC855	ACACACACACACACACYT	UBC857	ACACACACACACACACYG
UBC864	ATGATGATGATGATGATG	UBC881	GGGTGGGGTGGGGTG
UBC889	DBDACACACACACACAC		

*RAPD*			
S7	GGTGACGCAG	S17	AGGGAACGAG
S21	CAGGCCCTTC	S43	GTCGCCGTCA
S58	GAGAGCCAAC	S121	ACGGATCCTG
S160	AACGGTGACC	S201	GGGCCACTCA
S256	CTGCGCTGGA	S412	GGGACGTTGG
S1403	TGGCGCACAC	S1409	GGGCGACTAC
SBS-A16	ACCTGGACAC	OPA-4	AATCGGGCTG
OPA-10	GTGATCGCAG	OPB-8	GTCCACACGG
RAPD-1	CCAGCCGAAC	RAPD-5	AGCGCCATTG
RAPD-7	ACCCGGTCAC		

*Note*. R = (A/G), Y = (C/T), and D = (A/G/T); ^a^average of the column.

**Table 3:** The characteristics of the bands generated by RAPD primers.

Primer	TF	PF	PFR (%)	PIC	RP	EMR	MI
S7	19	15	78.95	0.33	25.48	11.84	3.90
S17	18	16	88.89	0.46	19.30	14.22	6.60
S21	16	14	87.50	0.36	20.43	12.25	4.43
S43	21	18	85.71	0.45	23.13	15.43	6.93
S58	14	12	85.71	0.20	22.43	10.29	2.04
S121	14	12	85.71	0.34	18.52	10.29	3.48
S160	18	12	66.67	0.38	22.35	8.00	3.03
S201	20	13	65.00	0.30	28.17	8.45	2.50
S256	15	13	86.67	0.41	17.57	11.27	4.67
S412	15	13	86.67	0.34	19.65	11.27	3.89
S1403	19	13	68.42	0.36	24.43	8.89	3.18
S1409	18	15	83.33	0.39	21.83	12.50	4.92
SBS-A16	14	12	85.71	0.34	18.61	10.29	3.45
OPA-4	10	7	70.00	0.24	15.13	4.90	1.19
OPA-10	19	18	94.74	0.49	19.22	17.05	8.43
OPB-8	15	13	86.67	0.38	18.52	11.27	4.31
RAPD-1	16	13	81.25	0.40	19.04	10.56	4.28
RAPD-5	15	11	73.33	0.37	18.87	8.07	2.99
RAPD-7	19	17	89.47	0.37	24.00	15.21	5.60
Average	16.58	13.53	81.60^a^	0.36^a^	20.88^a^	11.16^a^	4.20^a^
Minimum	10	7	65.00	0.20	15.13	4.90	1.19
Maximum	21	18	94.74	0.49	28.17	17.05	8.43
Total	315	257					

*Note*. ^a^Average of the column. TF, total number of fragments; PF, number of polymorphic fragments; PFR, polymorphic fragments ratios (%); PIC, polymorphism information content; RP, resolving power; EMR, effective multiplex ratio; MI, marker index.

**Table 4:** The characteristics of the bands generated by ISSR primers.

Primer	TF	PF	PFR (%)	PIC	RP	EMR	MI
UBC807	19	15	78.95	0.37	23.83	11.84	4.42
UBC810	18	15	83.33	0.50	18.00	12.50	6.25
UBC826	11	9	81.82	0.41	12.96	7.36	3.03
UBC827	12	11	91.67	0.56	10.52	10.08	5.66
UBC829	8	8	100.00	0.53	7.57	8.00	4.22
UBC834	16	14	87.50	0.45	17.48	12.25	5.56
UBC836	13	9	69.23	0.34	17.13	6.23	2.13
UBC846	15	15	100.00	0.51	14.61	15.00	7.70
UBC847	11	10	90.91	0.39	13.39	9.09	3.56
UBC848	13	11	84.62	0.49	13.22	9.31	4.58
UBC855	10	9	90.00	0.66	6.78	8.10	5.35
UBC857	13	12	92.31	0.45	14.35	11.08	4.96
UBC864	14	13	92.86	0.43	15.91	12.07	5.21
UBC881	16	15	93.75	0.44	18.00	14.06	6.15
UBC889	15	12	80.00	0.46	16.17	9.60	4.42
Average	13.60	11.87	87.80^a^	0.47^a^	14.66^a^	10.44^a^	4.88^a^
Minimum	8	8	69.23	0.34	6.78	6.23	2.13
Maximum	19	15	100.00	0.66	23.83	15.00	7.70
Total	204	178					

*Note*. ^a^Average of the column. TF, total number of fragments; PF, number of polymorphic fragments; PFR, polymorphic fragments ratios (%); PIC, polymorphism information content; RP, resolving power; EMR, effective multiplex ratio; MI, marker index.

**Table 5:** Comparative analysis of genetic variability in *Prunus* L. landraces using ISSR, RAPD, and combined data.

Analysis	ISSR	RAPD	ISSR + RAPD
No. of primers	15	19	34
Total no. of fragments	204	315	519
No. of polymorphic fragments	178	257	435
Average of total fragments	13.60	16.58	15.09
Average of polymorphic fragments	11.87	13.53	12.70
Polymorphism fragments ratios	87.80	81.60	84.70
Polymorphism information content	0.47	0.36	0.42
Resolving power	14.66	20.88	17.77
Effective multiplex ratio	10.44	11.16	10.80
Marker index	4.88	4.20	4.54
Dice's similarity coefficient	0.558–0.892	0.584–0.860	0.597–0.865
Average Dice's similarity coefficient	0.748	0.762	0.756

**Table 6:** The similarity matrix of the landraces using Dice's coefficient based on the ISSR and RAPD bands.

No.	P1	P2	P3	P4	P5	P6	P7	P8	P9	P10	P11	P12	P13	P14	P15	P16	P17	P18	P19	P20	P21	P22	P23	P24	P25
P1	1																								
P2	0.928	1																							
P3	0.886	0.949	1																						
P4	0.763	0.764	0.753	1																					
P5	0.776	0.805	0.797	0.759	1																				
P6	0.741	0.770	0.755	0.751	0.795	1																			
P7	0.709	0.737	0.730	0.741	0.736	0.786	1																		
P8	0.674	0.695	0.699	0.695	0.732	0.709	0.699	1																	
P9	0.697	0.707	0.699	0.776	0.705	0.716	0.757	0.676	1																
P10	0.693	0.714	0.714	0.703	0.716	0.709	0.653	0.668	0.653	1															
P11	0.639	0.630	0.630	0.660	0.678	0.643	0.614	0.699	0.668	0.680	1														
P12	0.657	0.678	0.682	0.670	0.714	0.703	0.689	0.816	0.701	0.662	0.709	1													
P13	0.641	0.628	0.635	0.612	0.672	0.649	0.628	0.651	0.685	0.597	0.732	0.707	1												
P14	0.666	0.691	0.699	0.699	0.732	0.747	0.722	0.722	0.695	0.718	0.714	0.716	0.647	1											
P15	0.720	0.722	0.714	0.695	0.728	0.724	0.664	0.684	0.653	0.865	0.657	0.662	0.620	0.745	1										
P16	0.720	0.722	0.722	0.664	0.682	0.712	0.749	0.668	0.722	0.657	0.649	0.685	0.697	0.687	0.687	1									
P17	0.641	0.643	0.651	0.631	0.653	0.676	0.651	0.612	0.705	0.612	0.682	0.660	0.734	0.647	0.639	0.682	1								
P18	0.728	0.749	0.757	0.710	0.778	0.801	0.687	0.714	0.672	0.726	0.660	0.693	0.647	0.707	0.761	0.714	0.647	1							
P19	0.699	0.720	0.720	0.716	0.741	0.699	0.697	0.712	0.697	0.724	0.678	0.714	0.653	0.712	0.736	0.693	0.626	0.759	1						
P20	0.695	0.728	0.739	0.705	0.745	0.726	0.682	0.716	0.685	0.759	0.666	0.703	0.633	0.728	0.755	0.689	0.618	0.770	0.830	1					
P21	0.722	0.732	0.747	0.724	0.745	0.722	0.716	0.712	0.689	0.716	0.658	0.726	0.645	0.716	0.743	0.724	0.649	0.770	0.834	0.795	1				
P22	0.672	0.678	0.685	0.697	0.707	0.687	0.728	0.682	0.755	0.655	0.666	0.691	0.695	0.685	0.655	0.682	0.695	0.678	0.680	0.699	0.703	1			
P23	0.697	0.714	0.718	0.699	0.755	0.685	0.691	0.710	0.714	0.718	0.668	0.705	0.643	0.691	0.710	0.657	0.643	0.699	0.832	0.801	0.797	0.724	1		
P24	0.695	0.701	0.709	0.689	0.737	0.691	0.697	0.701	0.689	0.712	0.666	0.695	0.645	0.693	0.701	0.693	0.645	0.693	0.807	0.772	0.803	0.699	0.840	1	
P25	0.712	0.710	0.710	0.699	0.724	0.716	0.676	0.710	0.676	0.726	0.703	0.685	0.643	0.714	0.737	0.668	0.643	0.726	0.782	0.755	0.774	0.689	0.761	0.759	1

*Note*. The bold values indicate the maximum and minimum genetic similarity values among the landraces.

**Table 7:** Typical band patterns amplified by ISSR and RAPD markers.

Primer	Approximate size of typical band (bp)	Identified varieties
UBC810	600	P1, P2, P3
UBC829	1600	P23
UBC834	400	P1, P2, P3
UBC836	1800	P1, P2, P3
UBC847	1000	P6
UBC847	550	P7
UBC848	1800	P8
UBC855	720	P7
UBC857	1700	P9
UBC881	800	P5
UBC889	350	P16
S17	250	P4
S43	400	P17
S1403	800	P17
RAPD-1	780	P12
RAPD-5	1100	P25

## Data Availability

The data that support the findings of this study are available on request to the corresponding author.

## Abbreviations

RAPD: Random amplified polymorphic DNA
SSR: Simple sequence repeat
ISSR: Intersimple sequence repeat
AFLP: Amplified fragment length polymorphism
CTAB: Cetyl trimethylammonium bromide
SM: Similarity matrix
SI: Similarity index
NTSYS: Numerical taxonomy multivariate analysis system
UPGMA: Unweighted pair group method with arithmetic mean algorithm
PIC: Polymorphism information content
RP: Resolving power
EMR: Effective multiplex ratio
MI: Marker index
TF: Total number of fragments
PF: Polymorphic fragments
PFR (%): Polymorphic fragments ratios (%).
